# *Akkermansia muciniphila* suppressing nonalcoholic steatohepatitis associated tumorigenesis through CXCR6^+^ natural killer T cells

**DOI:** 10.3389/fimmu.2022.1047570

**Published:** 2022-12-01

**Authors:** Tao Li, Xinlong Lin, Binhai Shen, Wujian Zhang, Yangyang Liu, Hongbin Liu, Ye Wang, Lijun Zheng, Fachao Zhi

**Affiliations:** ^1^ Guangdong Provincial Key Laboratory of Gastroenterology, Department of Gastroenterology, Institute of Gastroenterology of Guangdong Province, Nanfang Hospital, Southern Medical University, Guangzhou, China; ^2^ Department of General Surgery of the First Affiliated Hospital of Heilongjiang University of Traditional Chinese Medicine, Haerbin, China; ^3^ Guangzhou ZhiYi Biotechnology Co. Ltd., Guangzhou, China

**Keywords:** cancer progression, *Akkermansia muciniphila*, tumor immune surveillance, nonalcoholic fatty liver disease, hepatocellular carcinoma - metabolic syndrome - non-alcoholic fatty liver disease (NAFLD) - non-alcoholic steatohepatitis - NASH-HCC

## Abstract

**Introduction:**

Gut microbiota plays a crucial role in the development and progression of nonalcoholic steatohepatitis (NASH) and associated hepatocellular carcinoma (HCC). *Akkermansia muciniphila* was reported to inhibit inflammation-associated cancer in the intestine. The anti-NASH ability of *A. muciniphila* has recently been found. Thus, we were to investigate whether supplementation of *A. muciniphila* could prevent NASH-associated HCC.

**Methods:**

In a model we called STAM, male C57BL/6J mice were subcutaneously injected with 200 µg streptozotocin at 4 days after birth, and fed with high-fat diet at 4 weeks of age to induce NASH-associated HCC. Faeces from mice and patients with NASH-related HCC were collected for 16S rRNA sequencing. STAM mice were orally administered either saline or *A. muciniphila* twice a day starting at 4 or 10 weeks of age. The effects of *A. muciniphila* on the immune responses were also evaluated.

**Results:**

Patients and mice with NASH-related HCC showed significantly reduced gut *A. muciniphila* in comparison to healthy controls. Administration of breast milk-isolated *A. muciniphila* (AM06) but not feces-isolated *A. muciniphila* (AM02) could improve NASH severity. Interestingly, breast milk-isolated *A. muciniphila* treatment suppressed the progression of NASH to HCC, accompanied with an increased hepatic CXCR6^+^ natural killer T (NKT) cell and decreased macrophage infiltration. The antitumor ability of *A. muciniphila* was not evident in NKT cell-deficient mice (CD1d^-/-^ and CXCR6^-/-^). *In vitro*, *A. muciniphila* promoted the killing of hepG2 cells by NKT cells.

**Discussion:**

Our study will provide the rationale for the application of *A. muciniphila* to treat NASH and for the prevention of its progression to HCC.

## Background

With the sharply improvement of living standards, nonalcoholic fatty liver disease (NAFLD) with an incidence of 25% is emerging as the most common chronic liver disease worldwide (1, 2). NAFLD includes a spectrum of liver diseases ranging from nonalcoholic fatty liver to nonalcoholic steatohepatitis (NASH), cirrhosis, and hepatocellular carcinoma (HCC) (3, 4). When nonalcoholic fatty liver develops to NASH, the risk for developing to HCC sharply increases (5). NASH can directly progress to HCC without cirrhosis as a necessary intermediate process (3) and is an independent predictor of poor outcome in patients with NAFLD (6). In addition, NASH could superimpose viral hepatitis, the leading etiology of HCC, to progress to HCC (7). HCC has become the third leading cause of death in patients with cancer worldwide. However, therapeutic methods that could slow or even reverse the progression of NAFLD have not been well defined, and no effective drugs for NASH are available. Moreover, no strategies for preventing NAFLD/NASH-associated HCC transition has been established. Thus, effective therapeutic strategies to inhibit nonalcoholic fatty liver progress to NASH and prevent its transition to HCC are urgently needed.

Intestinal microbiome determines the development of NAFLD and NAFLD-associated HCC via gut-liver axis (8–10). Therefore, intestinal microbiota has gradually become a novel therapeutic target for NAFLD and preventive approach for associated HCC (10). Probiotic supplementation could ameliorate NAFLD (11, 12), while relatively little is known about the effects of probiotic administration on the progression of NAFLD to HCC. Though probiotic administration has been proven to suppress implanted and chemically induced HCC growth in mice (13, 14), whether probiotics can suppress NAFLD-associated HCC has not been investigated. *Akkermansia muciniphila* (AKK), attracting growing interest for its potential as a promising next-generation probiotic (15), was most recently found to have anti-NAFLD activity and could be a potential agent for clinical intervention in NAFLD (11, 12). *A. muciniphila* was markedly reduced in patients with NAFLD-associated HCC and was inversely correlated with inflammatory markers (16, 17). However, whether *A. muciniphila* recovery can suppress the progression of NAFLD to HCC remains unexplored.

*A. muciniphila* is a gram-negative anaerobic bacterium in human intestinal tract (13). In addition to its beneficial significances in improving metabolic disorder (18), accumulating evidences have indicated that *A. muciniphila* could blunt tumorigenesis in the intestine by promoting tumor immune surveillance (19, 20). A recent study found that *A. muciniphila* could attenuate inflammation and blunt inflammation-induced tumorigenesis in colon by regulating the anti-tumor ability of CD8^+^ T cells (20). NASH-associated HCC is a typical inflammation-associated cancer and is closely associated with gut microbiota profile (8, 11). Therefore, we speculated that supplementation of *A. muciniphila* could reduce steatohepatitis in NASH and prevent NASH to HCC progression by promoting hepatic immune surveillance. Natural killer T (NKT) cells are predominantly found in liver and play a vital role in the pathogenesis and progression of liver diseases (21). Probiotics could promote the hepatic homing of NKT cells to improve high-fat diet (HFD)-induced hepatic steatosis (22). Thus, we investigated whether *A. muciniphila* could promote hepatic accumulation of NKT cells and exert anti-HCC effects through NKT cells.

Murine NASH-HCC were induced by neonatal injection of streptozotocin (STZ) and followed by HFD, called STAM (23). The present study showed that oral supplementation of *A. muciniphila* attenuated NASH and its progression to HCC. Further investigation found that *A. muciniphila* increased infiltration of NKT cells but decreased macrophages in livers of STAM mice. More importantly, the antitumor ability of *A. muciniphila* was abolished in NKT-deficient mice, indicating that *A. muciniphila* blunt NASH to HCC progression through NKT cells. Taken together, this study highlight the potential of *A. muciniphila* in treating NAFLD and preventing NASH-HCC progression.

## Methods

### Antibodies and reagents

FITC anti-CD3 antibody (ab91493), Alexa Fluor® 647 anti-CD8 alpha antibody [EPR21769] (ab237365), PE anti-CD1d antibody [1B1] (ab93508), PE anti-mouse F4/80 (ab123110) for flow cytometry and anti-F4/80 antibody (Alexa Fluor® 488)-ab204266 for immunofluorescence were obtained from Abcam. Mouse CXCR6 APC-conjugated Antibody (# FAB2145A) was from R&D SYSTEMS. CD1d Tetramer a-GalCer Loaded (R-PE) (Proimmune, Oxford, UK) was from Shanghai Yes Service Biotech Inc. Mouse Alanine Aminotransferase (ALT/GPT) ELISA Kit (CSB-E16539m) and Mouse Aspartate Aminotransferase (AST) ELISA kit (CSB-E12649m) were from Huamei Biological Engineering Co., Ltd. Annexin V-FITC/PI Apoptosis Detection Kit I was from Beijing Solarbio Science and Technology Co.,Ltd. 60 kcal% high-fat diet (HFD, Research Diets, D12492) was from Hangzhou Xincheng Biotechnology Co., Ltd. Streptozotocin (Sigma-Aldrich, MO, USA) was from Shanghai Haoyang Biotechnology Co., Ltd. DMEM, penicillin-streptomycin liquid (Solarbio) and trypsin (Beyotime) were from Guangzhou Shuoheng Biotechnology Co., Ltd. Fluorescein isothiocyanate (FITC)-labelled *A. muciniphila* probe (FITC-CCTTGCGGTTGGCTTCAGAT) was from GENERAY Biotechnology (Shanghai, China). Glycolipid a-galactosyl ceramide (a-GalCer, KRN7000) was from MedChemExpress.

### Patients and sample collection

Patients who were diagnosed with NAFLD or NAFLD-HCC were enrolled in the study after obtaining their informed consent. Patients were clinically diagnosed with liver diseases, meeting the criteria of NAFLD: fatty liver from imaging (ultrasound or tomography) or from biopsy according to NAFLD activity score. NAFLD-HCC was diagnosed according to international guidelines (24).

### Animal model

Wild type (WT) 14-day pregnant specific pathogen-free (SPF) C57BL/6J mice CXCR6-knockout and CD1d-knockout mice were from Cyagen Biosciences Inc. At 4 days after birth, male mice were subcutaneously injected with 200 µg STZ for one time and then fed with 60 kcal% HFD ad libitum starting from 4 weeks of age to induce NASH-HCC. Control group were healthy male mice that kept on a normal diet without any treatment. Every cage included five to six mice as a group under conditions of controlled temperature (23 ± 3°C), humidity (50% ± 5%) and lighting (12/12-h light-dark cycle). Mice were free to get food and water. Before experiments, all mice were numbered. Each group included ten mice by randomly allocated number, but only six to eight mice were alive at the end point of the experiments because of liver disease. Besides, mice that have prematurely died or animals with weak vital signs were also excluded. All animal procedures were performed in accordance with the ‘‘Guide for the Care and Use of Laboratory Animals’’ prepared by the National Academy of Sciences and published by the National Institutes of Health (revised 1985). The study conformed to the ethical guidelines of the Declaration of Helsinki and approval by the Ethics Committee. Mice were euthanized at 10 or 20 weeks of age and the liver tissues were collected for various analyses.

### Experimental design

Mice were orally administered with phosphate-buffered saline (PBS) (STAM group) or 1.0×10^9^ CFU *A. muciniphila* twice a day starting at 4 weeks of age. Four-week-old mice were euthanized for baseline analysis. At the end point, blood samples were obtained from the right atrium *via* cardiac puncture, and their livers were excised. To explore the influences of *A. muciniphila* gavage on the process of NASH-to-HCC transition, another two groups of mice were treated with either PBS or 1.0×10^9^ CFU *A.muciniphila* twice a day from 10 weeks of age to 20 weeks of age. To figure out the role of NKT cells in hepatocarcinogenesis, CD1d^−/−,^ CXCR6^−/−^ or WT mice were treated with either PBS or 1.0×10^9^ CFU *A.muciniphila* twice a day from 10 to 20 weeks of age. As a positive control of NKT cell activation, mice were injected i.v. *via* the lateral tail vein with a-GalCer (50 mg per kg). Only the co-authors who were responsible for keeping mice and data analysis were aware of the group allocation. All experimental designs and protocols were drawn in **Figures 3A**, **4A**, **6A**.

### Cultivation of *A. muciniphila*

A. *muciniphila* isolated from breast milk (AM06) and feces (AM02) were kindly presented by Guangzhou Zhiyi Biotechnology Co., LTD. *A. muciniphila* was cultured in brain heart infusion broth under strict anaerobic conditions at 37°C. Before the administration, *A. muciniphila* was diluted in sterile PBS.

### 16S rRNA sequencing and analysis

Sequencing was conducted as previously described (25). DNA was extracted from collected stool samples and 16S V3-V4 region amplification were performed on the liquid hand ling robots. After genomic DNA extraction, PCR amplification, the DNA amplicons are linearized as single-stranded. Adding modified DNA polymerase and four fluorescently labeled dNTP to only one base in each cycle. Scan the reaction plate surface by laser and read the nucleotide species polymerized in the first round of reaction of each template sequence.

### Quantification of faecal *A. muciniphila* by qPCR

*A. muciniphila* sequences: forward CAGCACGTGAAGGTGGGGAC and reverse CCTTGCGGTTGGCTTCAGAT. E. coli containing target gene plasmids in the cloned library were selected for 37°C shake flask culture. Plasmids were extracted using the Axygen plasmid miniprep Kit (Axygen) and the concentration was determined using Qubit 3.0 (Life Biotech). After calculation of the plasmid copy number, the standard curve was obtained by diluting the plasmid DNA in a 10-fold gradient series to amplify the standard sample together with the sample to be tested. Finally, The number of gene copies in the sample was calculated from the resulting standard curve and finally analyzed in gene copy number per μl.

### Flow cytometry

Cells were washed with PBS and resuspended in 100 μl flow cytometry staining buffer. After that, cells were labeled with the indicated antibodies for 15 min at 4°C in the dark room. The following markers were used to identify different immune cell subsets: CD3^+^CD8^+^ for CD8^+^ T cells, NKT cells were stained with anti-CD3 and anti-CD1d-tetramer-PE loaded with a-GalCer, and CD11b^+^F4/80^+^ for macrophages (including monocytes and kupffer cells). Absolute numbers were calculated by multiplying frequencies obtained from flow cytometry by the total live mononuclear cell count, and then divided by liver weight. Flow cytometry was performed on BD LSRFortessa platform and results were analyzed using FlowJo software version 10.6.2. Dead cells were excluded by using live/dead fixable near-IR dead cell staining kit (ThermoFisher scientific).

### Histological analysis

A part of the liver samples from mice were fixed in 4% paraformaldehyde solution for 24 to 48 hours, after that, samples were embedded in paraffin for reservation. Paraffin sections were stained with hematoxylin and eosin (HE). Using HE-stained samples, we calculated the NAFLD activity score (NAS) by combining the grades of steatosis (0-3), inflammation (0-3), and hepatocyte ballooning (0-2) (26). The histological scores of mice were assessed by two pathologists in a double-blind manner. For immunostaining, liver samples from mice were embedded in opti-mum cutting temperature compound. The endogenous enzymes of 8-μm-thick sections were then inactivated and antigens were thermally repaired. After that, slices were incubated in the primary antibodies (rat anti-mouse F4/80) at 4°C overnight and followed by corresponding secondary antibody (Boster BioEngineering, Wuhan, China).

### Coculture of NKT cells that isolated from mice with HepG2 cells

HepG2 cells were cultured in DMEM medium containing 10% fetal bovine serum at 37°C in a 5% CO2 humidified atmosphere. Splenocytes were isolated and cultured in fresh RPMI-1640 medium containing 20% FBS in the presence of 1.0×10^9^ CFU/100 µL *A. muciniphila* or *A. muciniphila*-culture medium for 24 hours. Then, NKT cells were isolated from the splenocytes by flow cytometry and cocultured with adherent HepG2 cells at 1:1 for 48 hours. After that, HepG2 cells were collected for detecting the mRNA levels of caspase-3 expression and apoptotic rate by Annexin V-FITC/PI Apoptosis Detection Kit I.

### Statistical analysis

Each group included 4 to 7 mice for *in vivo* experiments, and all the results were presented as mean ± SEM. Differences between two groups were analyzed by two-tailed Student’s t test using Graphpad Prism 5. P ≤ 0.05 was considered statistically significant.

Additional methods can be found online in **Supplementary Material**.

## Results

### Reduced faecal *A. muciniphila* abundance in NASH-HCC patients and the corresponding mouse models

**Figure 1:**
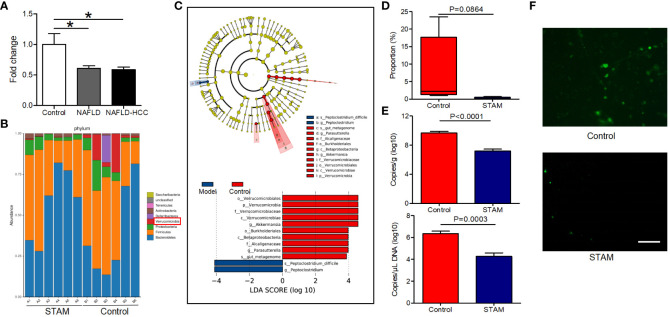
The intestinal abundance of *A muciniphila* was decreased in patients and mice with NAFLD-HCC (STAM at 20 weeks) **(A)** The relative abundance of *A muciniphila* in patients with NAFLD or NAFLD-HCC and healthy controls by qPCR (n=6). *P<0.05 by unpaired Student’s t test. **(B)** Comparison of the faecal microbiota between STAM at 20 weeks and controls at the species level by 16S rRNA sequencing. **(C)** LEfSe analysis of the faecal microbiota between the STAM at 20 weeks and healthy controls. Cladogram displays the taxonomic tree of differentially abundant taxa. Histogram represents the LDA scores of bacteria with significant differential abundance between the compared groups, identified by different colors. **(D)** The proportion of *Akkermansia* in the faecal microbiota was compared. **(E)** qPCR validation of the abundance of *A muciniphila* in STAM at 20 weeks and control. **(F)** FISH detection of *A. muciniphila* on the surface of the colon from STAM mice at 20 weeks of age and control mice. Data are presented as the mean ± SEM and were analysed by unpaired Student’s t test. NAFLD, non-alcoholic fatty liver disease; HCC, hepatocellular carcinoma; STAM, streptozotocin+high fat diet-treated mice.

Patients with NAFLD-related HCC showed a reduction in *A. muciniphila* in fecal microbiota (16). Here, we confirmed that *A. muciniphila* was reduced in the stools of patients with NAFLD and NAFLD-relaed HCC (**Figure 1A**). More importantly, we utilized the STAM model of murine NASH-HCC to confirm the reduction of *A. muciniphila* in NASH-HCC. This model is closely similar to human NASH with consistent progression to HCC in male mice. STAM developed to NAFLD-related HCC at the age of 20 weeks, then the stools were collected for16S rRNA sequencing. Compared to stools from healthy mice, stools from STAM showed significantly decreased abundance of *Verrucomicrobia* and *A. muciniphila* (the only representative member of the *Verrucomicrobia* phylum) (**Figures 1B, C**). Further, the proportion of *A. muciniphila* was reduced in STAM (**Figure 1D**; **Supplementary Figure 1**). The decreased abundance of *A. muciniphila* was confirmed by qPCR and FISH (**Figures 1E, F**). The diversity (Shannon index) and richness (Chao index) of the gut microbiota in STAM at 20 weeks were significantly reduced compared to those of healthy controls (**Supplementary Figure 2**).

### The successful establishment of STAM model

**Figure 2:**
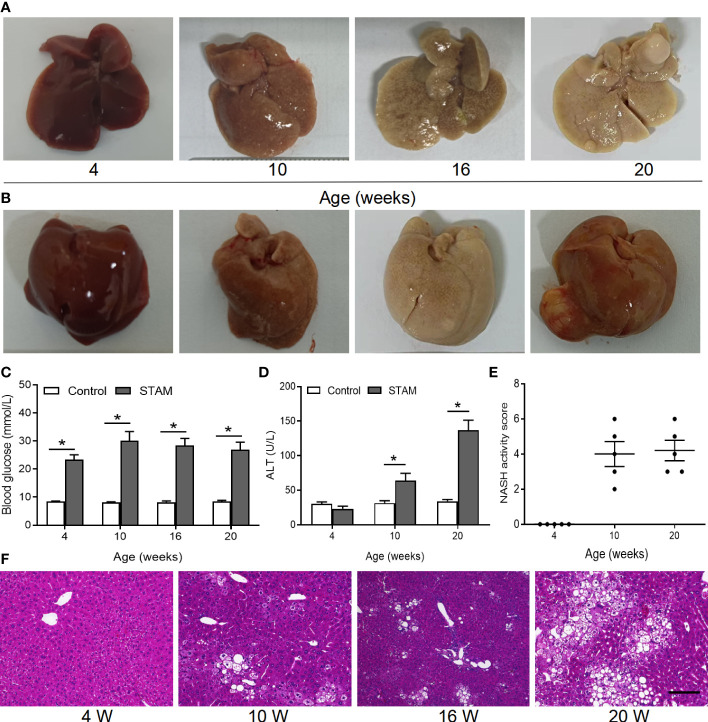
The STAM model of diabetes and high fat diet leading to NASH and HCC. **(A, B)** By 20 weeks, male mice develop numerous liver tumors (n=6). Macroscopic image of the **(A)** back and **(B)** front side of liver surface. The serum levels of blood glucose **(C)** and ALT which were detected by ELISA **(D)** of the STAM mice were elevated. **(E)** STAM mice progressed with increased NASH activity scores (NAS) from 10 weeks of age. **(F)** Paraffin-embedded and frozen mouse liver sections were stained with haematoxylin and eosin (H&E) to determine liver histology (Scale bars, 50 µm). ALT, alanine aminotransferase. *P<0.05 vs STAM group by unpaired Student’s t test.

Loss of *A. muciniphila* may lead to intestinal and liver inflammation, which contributes to the initiation and/or the progression of tumorigenesis (16, 19). To investigate whether supplementation of *A. muciniphila* could the alleviate liver inflammation and blunt NASH-associated HCC, we established a murine model of NASH-HCC called STAM (23). Macroscopically, all livers of STAM showed pale yellow color at 10 weeks, granular surface at 16 weeks, and tumor nodules at 20 weeks (**Figures 2A, B**). STAM mice had markedly elevated blood glucose levels (**Figure 2C**), increased serum alanine aminotransferase (ALT) levels (**Figure 2D**), and NASH activity scores (**Figure 2E**) that were evaluated microscopically (**Figure 2F**) from the age of 10 weeks.

### *A. muciniphila* alleviates the liver steatohepatitis

**Figure 3:**
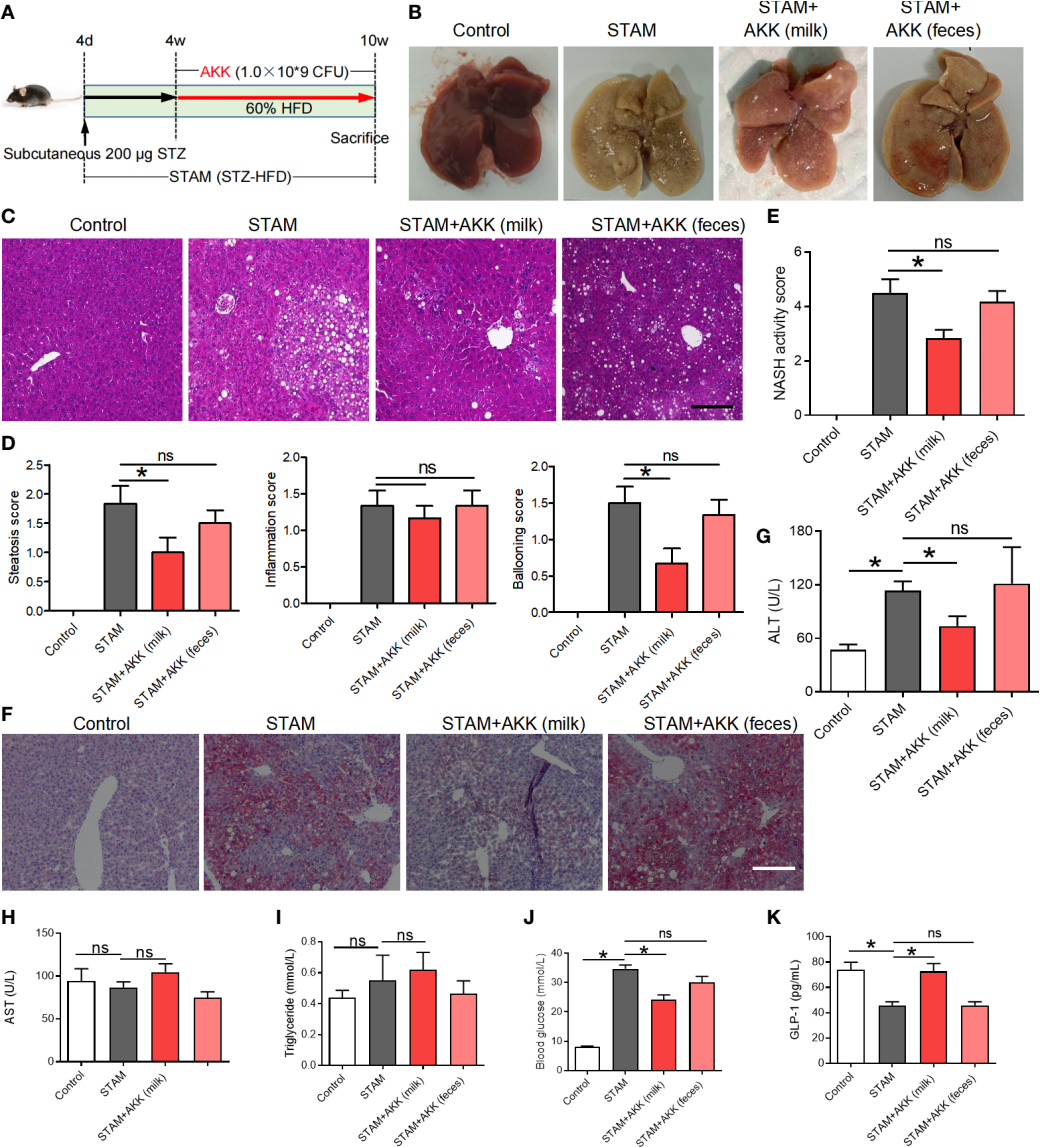
Supplementation with *A muciniphila* isolated from breast milk but not from stool improved nonalcoholic steatohepatitis in STAM mice. **(A)** Experimental design and protocol (n=6). **(B)** Representative macroscopic image of the liver surface and **(C)** histological images of liver tissues by H&E staining. Scale bars, 50 µm. **(D)** Steatosis, inflammation score and ballooning score were evaluated in the livers of STAM mice at 10 weeks of age. **(E)** The total NASH score was calculated by combining the sum of steatosis score, inflammation score, and ballooning score (n=6/group). **(F)** Oil-red stained liver tissues of STAM mice with or without *A muciniphila* adminstration. Scale bars, 50 µm. The serum ALT **(G)**, AST, triglyceride, blood glucose and GLP-1 **(H-K)** levels were measured. Data represent mean ± SEM of two pooled experiments. *P<0.05 vs STAM group by unpaired Student’s t test. ns, not significant. AKK, *Akkermansia muciniphila*; ALT, alanine aminotransferase; AST, aspertate aminotransferase; GLP-1, glucagon-like peptide-1.

To test whether supplementation of *A. muciniphila* could reduce liver inflammation, we simultaneously orally administered *A. muciniphila* twice a day when starting HFD in STAM model (**Figure 3A**). Both *A. muciniphila* isolated from breast milk and feces were used. At 10 weeks of age, mouse livers were harvested and subjected to histological analysis (**Figure 3B**). Hematoxylin and eosin staining revealed markedly increased vacuolated cells, confluent lipid droplets, inflammatory cell infiltration, and balloon-like structures in the livers of the STAM mice, which greatly disorganized the liver structure, while supplementation of milk-derived *A. muciniphila* but not feces-derived *A. muciniphila* could significantly suppressed these abnormalities (**Figures 3C, D**). Though the score of lobular inflammation had no difference, the score of steatosis (P<0.05) (**Figure 3D**), ballooning and NAS (NAFLD activity score) (P<0.05) in *A. muciniphila* (milk-derived) group were all lower than that in STAM model group (**Figure 3E**). Oil red O staining further validated that *A. muciniphila* administration attenuated fatty degeneration in the liver of NASH mice (**Figure 3F**). Furthermore, the level of serum ALT was significantly increased by *A. muciniphila* (milk-derived) administration in STAM mice (**Figure 3G**). But the aspertate aminotransferase (AST) and triglyceride levels had no change between the STAM and *A. muciniphila*-treated group (**Figures 1H, I**). *A. muciniphila* treatment decreased fasting blood glucose and increased fasting glucagon-like peptide-1 (GLP-1) levels in STAM mic at 10 weeks of age (**Figures 3J, K**). Taken together, these results indicated that *A. muciniphila* (milk-derived) gavage could markedly attenuate STAM-induced NASH development. Therefore, we only used breast milk-derived *A. muciniphila* in all the following experiments.

### *A. muciniphila* administration prevents NASH from progressing to liver cancer and biochemical profiles

**Figure 4:**
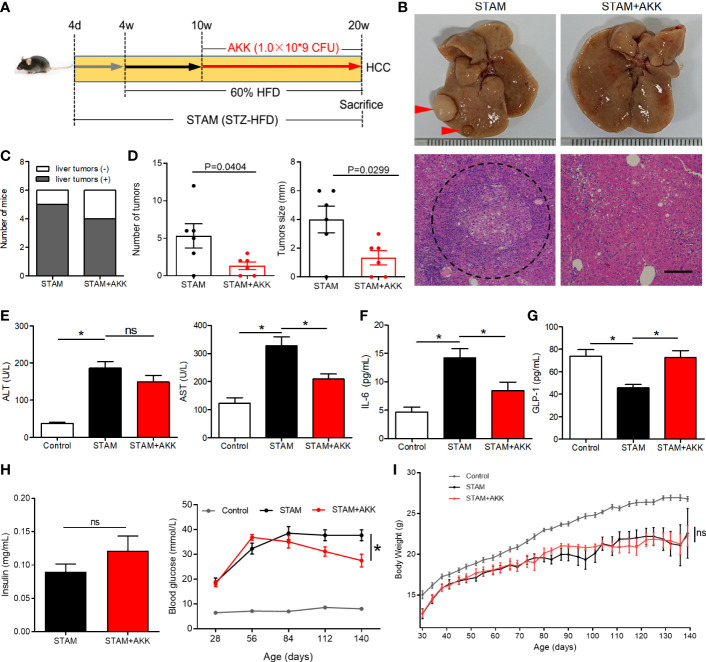
*A muciniphila* administration blunted hepatocarcinogenesis in STAM mice. **(A)** Experimental design and protocol. STAM mice were treated with *A muciniphila* or saline (Control) starting at 10 weeks of age for 10 weeks, and were killed at 20 weeks of age (n=6/group). **(B)** Macroscopic image of the liver surface (arrowheads: tumor) and histological images of liver tissues by H&E staining (dashed circle: tumor). Scale bars, 200 µm. **(C)** The total number of tumor nodules on the liver surface and the maximum diameter of the tumor nodules were compared between the groups. **(D)** The number of mice developed with liver tumor were counted. The serum levels of **(E)** ALT, AST, **(F)** GLP-1, **(G)** IL-6 and **(H)** insulin of the STAM mice (20 weeks of age) were measured. **(H)** The blood glucose were recorded every week. **(I)** The body weight was recorded every 4 days. Data represent mean ± SEM of two pooled experiments. *P<0.05 by unpaired Student’s t test. ns, not significant. STZ, streptozotocin; STAM, streptozotocin + high fat diet-treated mice; AKK, *Akkermansia muciniphila*; AST, aspertate aminotransferase; ALT, alanine aminotransferase; GLP-1, glucagon-like peptide-1.

To investigate the long-term effect of supplementation of *A. muciniphila* on the progression of NASH to HCC, we extended the treatment with an oral gavage of *A. muciniphila* (1.0×10^9^ CFU) starting at the time when STAM develop to NASH (10 weeks of age) to the full 20-week time course of the experiments (**Figure 4A**). STZ-HFD treatment severely disorganized liver structures characterized by steatosis, inflammation, and necrosis, which were partly recovered by *A. muciniphila* administration (**Figure 4B**). There was no statistical difference in the incidence of HCC with or without *A. muciniphila* administration in STAM mice. Of the 6 male mice, 5 in control STAM mice and 4 in *A. muciniphila*-treated STAM mice developed multiple liver tumors resembling HCC (**Figure 4C**). Macroscopically, the STAM mice supplemented with *A. muciniphila* developed only 1.3 ± 0.5 liver surface tumor nodules, while the control STAM littermates had only 5.3 ± 1.1 tumor nodules (p=0.0404) (**Figures 4B, D**). Moreover, tumors were also significantly smaller in *A. muciniphila*-treated group (**Figure 4D**). The elevated ALT levels in STAM were not improved by *A. muciniphila* administration, but aspertate aminotransferase (AST) levels tended decrease after *A. muciniphila* administration (**Figure 4E**). *A. muciniphila* administration might persistently alleviate the microenvironmental inflammation that induce HCC in NASH, for *A. muciniphila*-treated STAM showed lower IL-6 levels than those in control STAM (**Figure 4F**).

Physical and biochemical variables were recorded during *A. muciniphila* treatment in STAM mice. Supplementation of *A. muciniphila* increased GLP-1 levels and decreased fasting blood glucose in STAM (**Figures 4G, H**), while levels of insulin were not affected by *A. muciniphila* treatment (**Figure 4H**). STAM mice had decreased body weight and *A. muciniphila* treatment did not change the body weight (**Figure 4I**). It is known that intestinal microbiome regulates bile acid metabolism. Probiotics have been shown to reverse abnormal bile acid metabolism. To explored whether *A. muciniphila* administration could influence bile acid metabolism, hepatic bile acid composition was analysed. The primary bile acids (CA and T-β-MCA) were significantly raised, while secondary bile acids T-ω-MCA, taurodeoxycholic acid (TDCA), ω-MCA, and tauroursodeoxycholic acid (TUDCA) were reduced after *A. muciniphila* administration.

### *A. muciniphila* promoted CXCR6^+^ NKT cell accumulation and reduced macrophages in the liver

**Figure 5:**
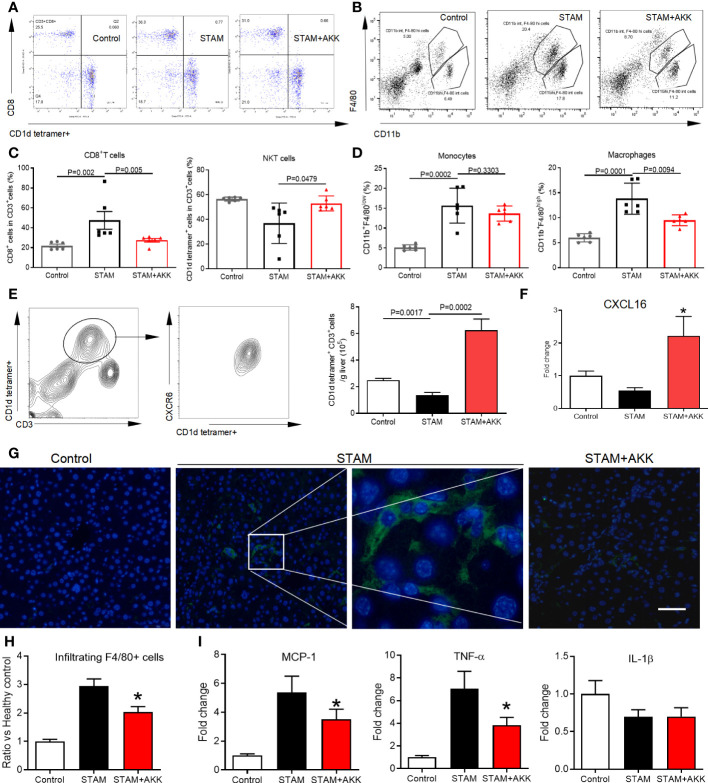
*A muciniphila* increases NKT cell but reduces macrophage infiltration and inflammatory cytokines in the livers of STAM. **(A)** Quantification of subgroups of T cells and **(B)** macrophages by flow cytometry. The percent of **(C)** CD8^+^ T cells, NKT cells, **(D)** monocytes and macrophages in the liver were evaluated. **(E)** Representative CXCR6 staining in hepatic NKT cells from three independent experiments. Relative mRNA levels of **(F)** CXCL16 in the liver were determined by real-time PCR and normalized to GAPDH. Data represent mean ± SEM of two pooled experiments (n=6/group). **(G)** Immunofluorescent staining for macrophages (Scale bars, 50 µm). **(H)** The infiltration macrophages in the liver were counted (vs healthy control group). **(I)** Relative mRNA levels of MCP-1, TNF-α and IL-1β in the liver were determined by real-time PCR and normalized to GAPDH. Data represent mean ± SEM of two pooled experiments (n=6/group). AKK, *Akkermansia muciniphila*. MCP-1, monocyte chemoattractant protein-1. *P<0.05 vs STAM group by unpaired Student’s t test.

To figure out the mechanisms by which *A. muciniphila* suppressed NASH-associated HCC, immune cell subsets in the liver were examined by flow cytometry (**Figures 5A, B** and **Supplementary Figure 4**). T lymphocytes and tumor-associated macrophage have been widely involved in regulating tumor immune surveillance (27). Here, hepatic NKT cells were prominently increased in the *A. muciniphila*-treated group, while CD8^+^ T cells were decreased (**Figure 5C**). The proportion of macrophages was decreased by *A. muciniphila* treatment, whereas no changes were found in monocytes (**Figure 5D**). The chemokine receptor CXCR6 expressed on activated T cells mediates their infiltration to chemokine ligand CXCL16 in the liver (28). As shown in **Figure 5E**, more than 90% NKT cells were CXCR6 positive. In line with increased NKT cell accumulation in the liver, *A. muciniphila* increased hepatic CXCL16 expression (**Figure 5F**). *A. muciniphila* treatment decreased macrophages accumulation in the livers of STAM (**Figures 5G, H**), accompanying with depressed inflammatory factors of monocyte chemoattractant protein-1 and TNF-α expression in the liver (**Figure 5I**).

### The anti-HCC ability of *A*. *muciniphila* depended on NKT cells

**Figure 6:**
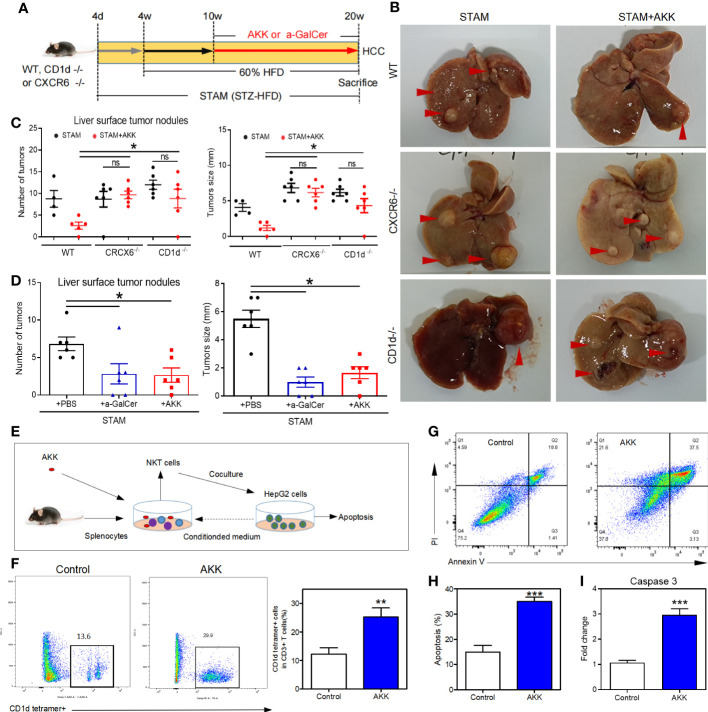
*A muciniphila* inhibit NASH-related HCC through NKT cells. **(A)** Experimental design and protocol. CD1d^−/−^ (n=6), CXCR6^−/−^ (n=6) or wild-type mice (n=4 or 5) were treated with *A muciniphila*, a-GalCer (postive control) or saline (placebo-control) for 10 weeks starting at 10 weeks of age twice a day. Mice were killed at 20 weeks of age. **(B)** Macroscopic images of the liver surface (arrowheads: tumor). **(C, D)** The total number of tumor nodules on the liver surface were counted and the maximum diameter of the tumor nodules in the liver were measured. **(E)** Experimental design and protocol of *in vivo* experiments. **(F)** The fraction of NKT cells in the splenocytes was cultured with HepG2 cell conditioned medium with or without *A muciniphila* for 72 hours. **(G, H)** The percentage of apoptotic HepG2 cells was measured by flow cytometry. Data represent mean ± SEM of two pooled experiments. **(I)** The mRNA expression of caspase 3 in HepG2 cells was detected by qPCR. *P < 0.05. **p<0.001, ***p<0.0001 by unpaired Student’s t test. STAM, streptozotocin + high fat diet-treated mice; AKK, Akkermansia muciniphila; ns, not significant.

To investigate whether *A. muciniphila* excert anti-tumor through NKT cell activation, we used CD1d-knockout mice (which completely lack NKT cells) (29) and CXCR6-knockout mice (which have a selective NKT deficiency in the liver). After the confirmation of hepatic deficiency of NKT cells, CD1d^-/-^ and CXCR6^-/-^ mice were induced to NASH-HCC with or without *A. muciniphila* gavage (**Figure 6A**). No reduction in liver surface tumor nodules and liver tumor size were found in CD1d^-/-^ and CXCR6^-/-^ mice after *A. muciniphila* gavage (**Figures 6B, C**). In addition, treatment with a-GalCer to potently activate NKT cells could also effectively reduce the number of liver tumor nodules and size in STAM (**Figure 6D**).

**Figure 7:**
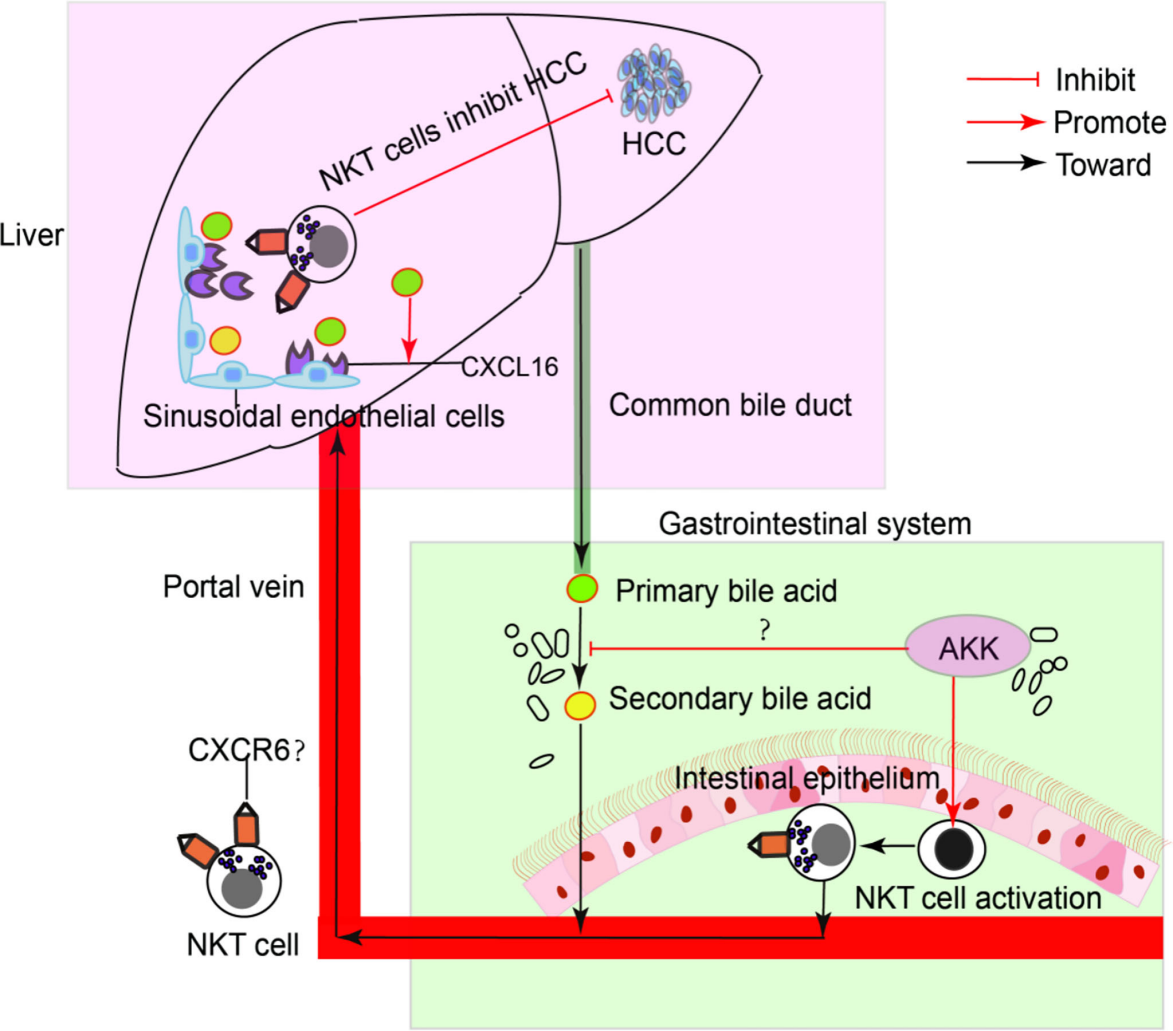
A model of the interaction of intestinal A. muciniphila with NKT cell to exert anti-tumor effects in liver *via* gut-liver axis.

To confirm the antitumour mechanism of *A. muciniphila*, splenocytes from normal mice were cultured with or without *A. muciniphila.* After that, NKT cells were isolated and cocultured with HepG2 cells (**Figure 6E**). *In vitro*, *A. muciniphila* markly raised the proportion of NKT cells in primary splenocytes cultured in HepG2 cell-conditioned medium (**Figure 6F**). Subsequently, NKT cells were isolated and cocultured with HepG2 cells. The purified NKT cells pretreated with *A. muciniphila* markedly increased the apoptosis of HepG2 cells (**Figures 6G, H**) and caspase 3 expression (**Figure 6I**). *In vitro* experiments found that *A. muciniphila* promoted the killing of HepG2 cells by NKT cells. In all, *A. muciniphila* inhibits tumorigenesis in liver by NKT cells as concluded in **Figure 7**.

## Discussion

The anti-tumor ability of probiotics to has been widely discovered. Efficacy of probiotics on tumor growth is mostly related to intestinal cancers. Yet, as the liver constantly exposed to gut-derived factors through the portal circulation, the influences of probiotics on liver cancer have also attracted the attention of researchers. However, studies regarding the application of probiotics to reduce the risk of developing to HCC remain limited. Here, we found the antitumor ability of a next-generation probiotic called *A. muciniphila* in the NAFLD-associated HCC microenvironment. Besides, our results give a novel insight into the mechanism by which *A. muciniphila* exert its systematic influences in the liver of NAFLD. In all, this study highlights the therapeutic potential of *A. muciniphila* in NASH and associated HCC.

*A. muciniphila* is a promising next-generation probiotics for its beneficial effects on maintaining systemic metabolism and immune stability (13). Recently, the antitumor activity of *A. muciniphila* have been discovered (30). *A. muciniphila* could maintain the sensitivity to immunotherapy, especially in anti-PD-1-based immunotherapy (31, 32). More importantly, *A. muciniphila* can not only directly inhibit cancer cell viability (33) but also enhance antitumor immune responses (20, 34). In the intestinal tract, *A. muciniphila* prevents colitis-associated colorectal cancer by promoting the activation of cytotoxic T lymphocytes in the colon (20). Gut microbiota modulates the systematic immune system. Regarding liver cancer, we found that *A. muciniphila* promoted hepatic NKT cells expanding to blunted steatohepatitis-associated hepatocarcinogenesis. Studies that reported probiotics could restore NKT cells supported our findings that *A. muciniphila* restored hepatic NKT cells (22, 35, 36). Both frequently residing in the liver, the role of NKT cells and macrophages in the pathogenesis of NAFLD-associated HCC remains controversial (37, 38). Decreased primary-to-secondary bile acid transition that regulated by gut microbiome remodeling could promote hepatic NKT cell infiltration to inhibit HCC (39). Consistently, we found that *A. muciniphila* reduced primary to secondary bile acid conversion accompanied with increased cytotoxic NKT cells to prevent hepatocarcinogenesis. Considering tumor-associated macrophages, *A. muciniphila* inhibited the proinflammatory macrophages which were reported to contribute to primary HCC progression (27).

Clinical trials based on targeting NKT cell activation (40) and direct infusion of NKT cells for the treatment of HCC have been recently reviewed (41). Most frequently residing in the liver, NKT cells can not only directly target hepatoma cells but also induce other immune cells to target hepatoma cells (42). In this study, we found that *A. muciniphila* could activate cytotoxic NKT cells and enhance the killing of hepatoma cells by NKT cells *in vitro*. *In vivo*, *A. muciniphila* promoted the infiltration of NKT cells in the liver. Therefore, *A. muciniphila* supplementation could blunt tumorigenesis in the liver. While NKT cells have well established therapeutic significances in cancers (40, 42), it has limited application and a high cost, which makes *A. muciniphila* a promising supplemental or alternative option for NKT cell-based therapy in cancers. *A. muciniphila*, a probiotic, could be added up to tumor immunotherapy to improve therapeutic efficacy and reduce cost of treatment without side effects.

STAM mice developed to NASH at 8 weeks of age and eventually develop to HCC at a rate of nearly 100% in males (23). With the similar clinical features of human HCC resulting from NASH, this mice model can be used for studying the treatment for NAFLD and prevention of NASH to HCC progression (23). When NAFLD developed to NASH, the risk for HCC significantly increased (20). Therefore, discovery of effective strategies to prevent the development and progression of NASH is urgently needed. In our preliminary experiments, STAM developed to NASH at the age of 10 weeks but not 8 weeks. Therefore, to investigate the effects of *A. muciniphila* on the progression of NASH to HCC, *A. muciniphila* was given from 10 weeks of age to the end point of 20 weeks of age. Recent studies have proven that feces-derived *A. muciniphila* administration could ameliorate fatty liver disease in obese mice *via* gut-liver axis. Interestingly, we found that treatment with breast milk-isolated *A. muciniphila* had superior anti-NASH activity than feces-derived *A. muciniphila*. More importantly, we extended the study and found *A. muciniphila* could prevent NASH from progressing to HCC. A clinical randomized controlled experiment has confirmed that oral supplementation of *A. muciniphila* can improve the blood markers (ALT and AST) for liver dysfunction and inflammation in humans (43). Thus, beyond the reasonable use of *A. muciniphila* for prevention of NASH onset, our results also support the application of *A. muciniphila* in patients with NASH to prevent them from progressing to HCC.

As the major etiologies of NAFLD, obesity and diabetes are two independent risk factors for NAFLD-HCC (44). The beneficial role of *A. muciniphila* in obesity and diabetes has been well established (15). *A. muciniphila* administration could reduce fat mass development, insulin resistance, and dyslipidemia to improve obesity and maintain glucose homeostasis in obese mice (44, 45). Thus, it is convincing that *A. muciniphila* administration could mitigate NAFLD-induced HCC, which was progressively induced in diabetic mice fed with HFD (STAM model). A recent study found that *A. muciniphila* could activate the secretion of the gastric intestinal hormone GLP-1 in the gut to improve obesity and maintain glucose homeostasis in mice fed with HFD (46). Here, we also found GLP-1 was elevated by *A. muciniphila* treatment in STAM. GLP-1 receptor agonist could ameliorate NASH and suppressed hepatocarcinogenesis in STAM (47). Therefore, it is reasonable that supplementation of *A. muciniphila* could improve NASH and prevent HCC in STAM.

We used two strains of AKK bacteria in preliminary experiments. One was isolated from feces, and the other was isolated from human breast milk. The average nucleotide identity (ANI) between AM02 and typical AKK strain (ATCC BAA-835) is 97.06%, The ANI between AM06 and ATCC BAA-835 is 99.99%. And the ANI between AM02 and AM06 is 97.06%. Commensal strains isolated from human breast milk have been proven safe and had beneficial effects (48, 49). Having confirmed the high safety and effectiveness of the breast milk-derived AKK, a patent for this strain is being applied. While previous studies uncovered the anti-NAFLD activity of AKK (14, 15), our study firstly demonstrated that AKK isolated from human breast milk has superior ability in preventing NAFLD than feces-derived AKK. However, we did not clarify why the two strains of AKK bacteria produced different effects.

Considering glycosphingolipids of gram-negative bacteria could promote NKT cell activation *in vivo* (9), *A. muciniphila* might carry glycolipid antigens that can be presented by CD1 to NKT cells. The frequency of NKT cells is high in mice, while the frequency of NKT cells is very low in humans. Besides, NKT cells in humans are functional versatile. Instead, among subsets of unconventional T cells, mucosa associated invariant T (MAIT) cells are arguably the most frequent in humans (50, 51). Therefore, in the human/clinical setting, vitamin B-derived antigens but not glycolipid antigens might be more important to mediate activation of unconventional T cells in humans by *A. muciniphila*. Further studies are needed to determine the interaction of *A. muciniphila* and MAIT and which components of *A. muciniphila* exert anti-HCC effects. Beyond the effects on the development of NASH, we found that *A. muciniphila* could inhibit NASH to HCC progression through NKT cells. *A. muciniphila* could regulate the immune system to defend against inflammation-related tumourigenesis. Therefore, *A. muciniphila* may be a promising therapeutic supplemental or alternative option for NASH and NASH-associated HCC.

## Data availability statement

The original contributions presented in the study are included in the article/**Supplementary Material**. The gene expression profiling data presented in the study are deposited in the National Library of Medicine repository, accession number PRJNA882939 (https://www.ncbi.nlm.nih.gov/sra/PRJNA882939).

## Ethics statement

The animal study was reviewed and approved by the Institutional Animal Care and Use Committee of Southern Medical University (K2019094).

## Author contributions

This study was designed by TL and FZ. TL, XL, BS and HL performed biologic evaluation, animal study and mechanism study. YW, LZ and YL provided reagents, materials and analysis tools. WZ collected the patient samples and provided data analysis. The manuscript was written by and TL. All authors contributed to the article and approved the submitted version.
