# Corrigendum: Akkermansia muciniphila suppressing nonalcoholic steatohepatitis associated tumorigenesis through CXCR6^+^ natural killer T cells

**DOI:** 10.3389/fimmu.2023.1139000

**Published:** 2023-02-22

**Authors:** Tao Li, Xinlong Lin, Binhai Shen, Wujian Zhang, Yangyang Liu, Hongbin Liu, Ye Wang, Lijun Zheng, Fachao Zhi

**Affiliations:** ^1^ Guangdong Provincial Key Laboratory of Gastroenterology, Department of Gastroenterology, Institute of Gastroenterology of Guangdong Province, Nanfang Hospital, Southern Medical University, Guangzhou, China; ^2^ Department of General Surgery of the First Affiliated Hospital of Heilongjiang University of Traditional Chinese Medicine, Haerbin, China; ^3^ Guangzhou ZhiYi Biotechnology Co. Ltd., Guangzhou, China

**Keywords:** cancer progression, *Akkermansia muciniphila*, tumor immune surveillance, nonalcoholic fatty liver disease, hepatocellular carcinoma - metabolic syndrome - non-alcoholic fatty liver disease (NAFLD) - non-alcoholic steatohepatitis - NASH-HCC

In the published article, there was an error in the legends for **Figure 1** to **Figure 6** as published. The order of **Figure 1** to **Figure 6** were all incorrect, and all their citations in the text were not listed in sequential order. The corrected figures and legends appears below.

**Figure 1:**
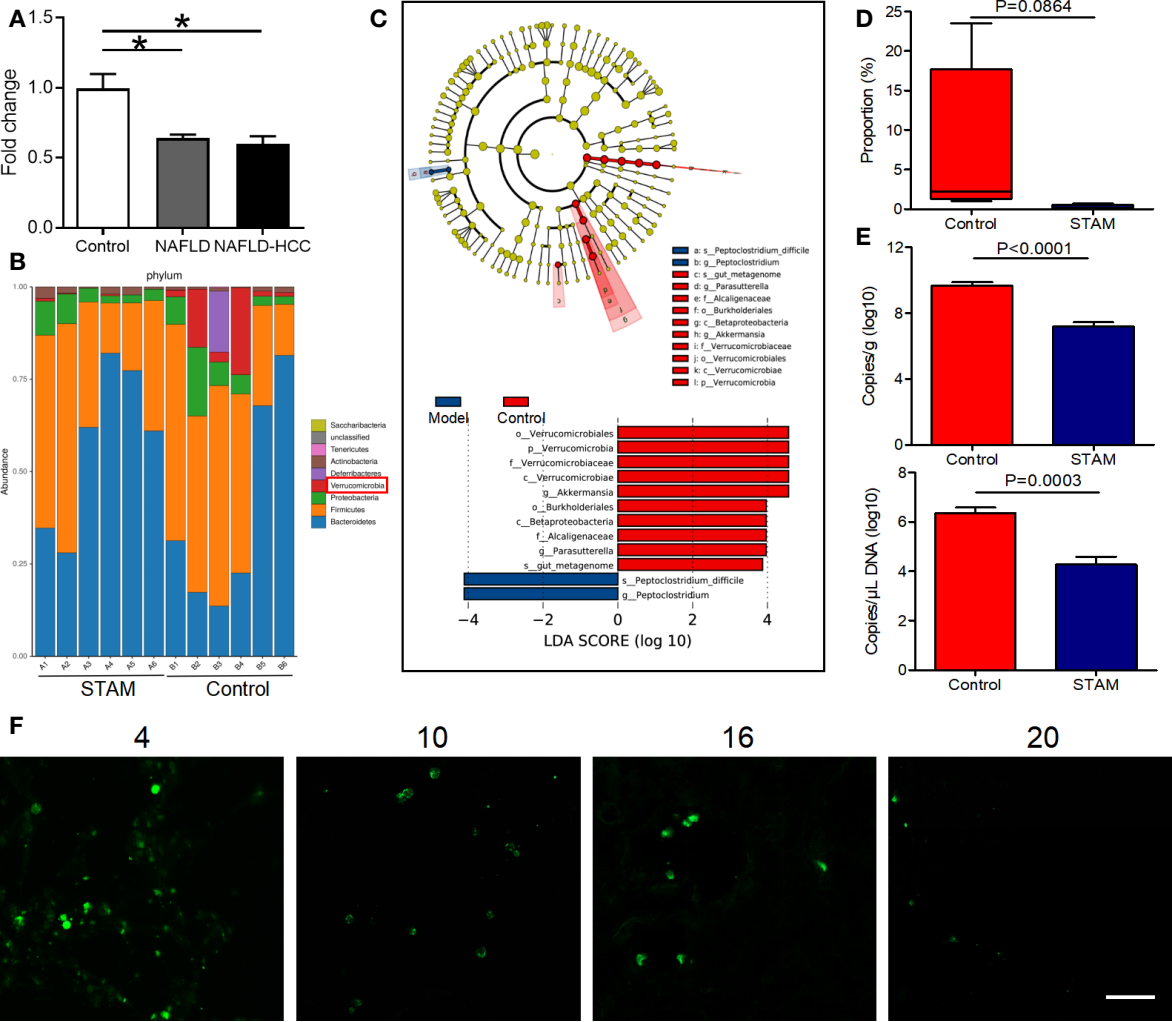
The intestinal abundance of *A muciniphila* was decreased in patients and mice with NAFLD-HCC (STAM at 20 weeks) **(A)** The relative abundance of *A muciniphila* in patients with NAFLD or NAFLD-HCC and healthy controls by qPCR (n=6). *P<0.05 by unpaired Student’s t test. **(B)** Comparison of the faecal microbiota between STAM at 20 weeks and controls at the species level by 16S rRNA sequencing. **(C)** LEfSe analysis of the faecal microbiota between the STAM at 20 weeks and healthy controls. Cladogram displays the taxonomic tree of differentially abundant taxa. Histogram represents the LDA scores of bacteria with significant differential abundance between the compared groups, identified by different colors. **(D)** The proportion of *Akkermansia* in the faecal microbiota was compared. **(E)** qPCR validation of the abundance of *A muciniphila* in STAM at 20 weeks and control. **(F)** FISH detection of *A muciniphila* on the surface of the colon from STAM mice at 4, 10, 16, 20 weeks of age. Data are presented as the mean ± SEM and were analysed by unpaired Student’s t test. NAFLD, non-alcoholic fatty liver disease; HCC, hepatocellular carcinoma; STAM, streptozotocin+high fat diet-treated mice.

In the published article, there was also an error with the figure citations. The figure citations in the text were not listed in sequential order in the **Results** part.

Corrections have been made through out the article to correct the figure citations.

**Figure 4** was corrected to **Figure 1**, **Figure 5** was corrected to **Figure 2**, **Figure 1** was corrected to **Figure 3**, **Figure 2** was corrected to **Figure 4**, **Figure 6** was corrected to **Figure 5**, **Figure 3** was corrected to **Figure 6**.

**Figure 2:**
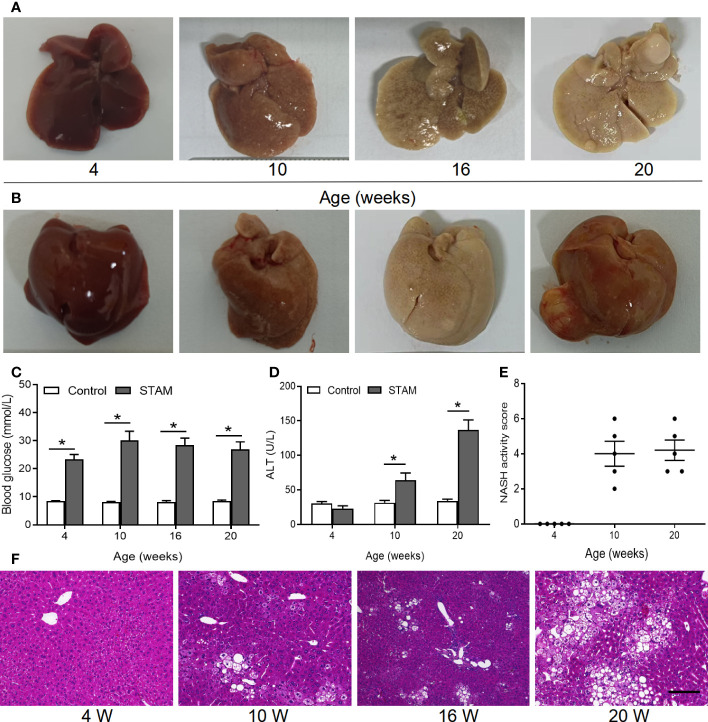
The STAM model of diabetes and high fat diet leading to NASH and HCC. **(A, B)** By 20 weeks, male mice develop numerous liver tumors (n=6). Macroscopic image of the **(A)** back and **(B)** front side of liver surface. The serum levels of blood glucose **(C)** and ALT which were detected by ELISA **(D)** of the STAM mice were elevated. **(E)** STAM mice progressed with increased NASH activity scores (NAS) from 10 weeks of age. **(F)** Paraffin-embedded and frozen mouse liver sections were stained with haematoxylin and eosin (H&E) to determine liver histology (Scale bars, 50 µm). ALT, alanine aminotransferase. *P<0.05 vs STAM group by unpaired Student’s t test.

**Figure 3:**
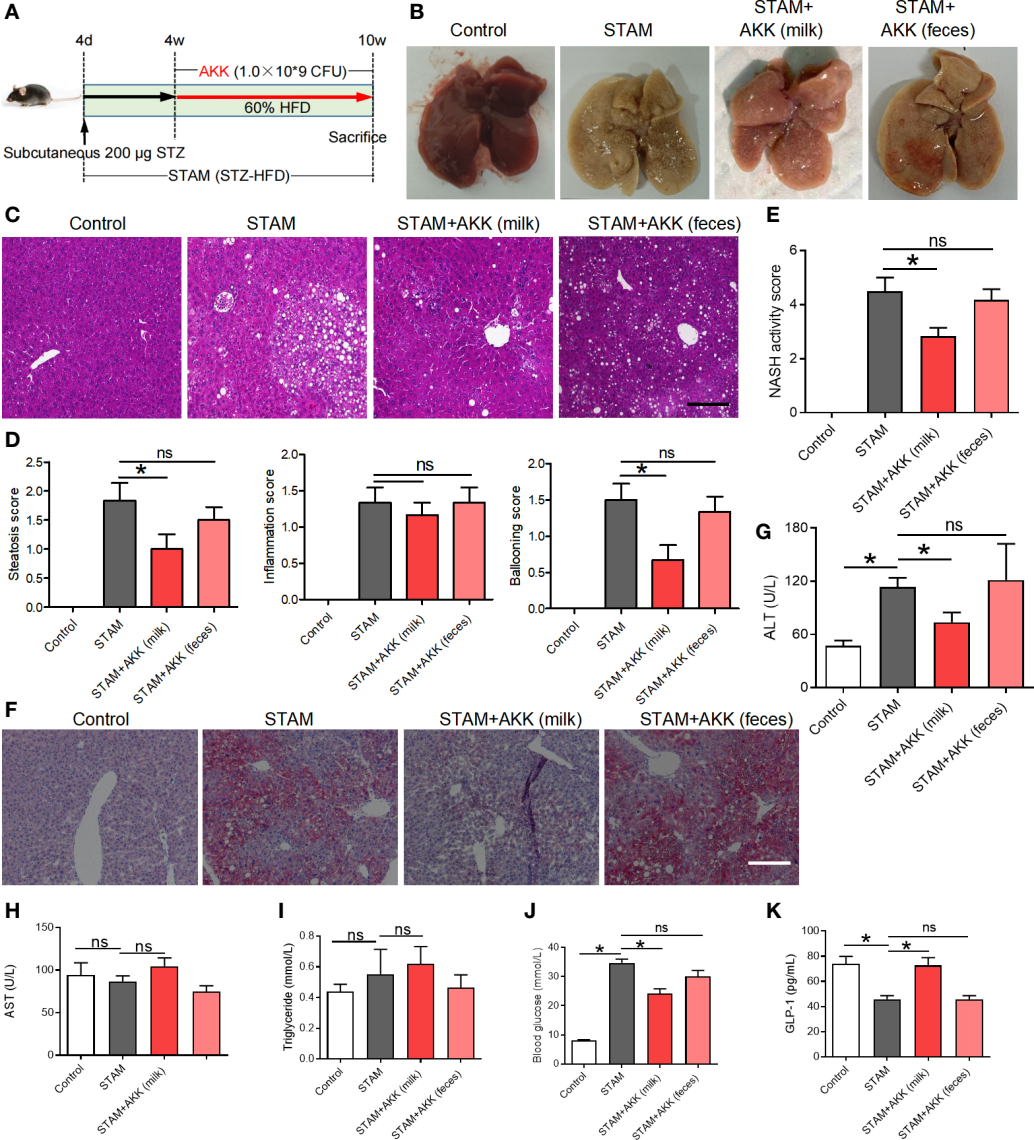
Supplementation with *A muciniphila* isolated from breast milk but not from stool improved nonalcoholic steatohepatitis in STAM mice. **(A)** Experimental design and protocol (n=6). **(B)** Representative macroscopic image of the liver surface and **(C)** histological images of liver tissues by H&E staining. Scale bars, 50 µm. **(D)** Steatosis, inflammation score and ballooning score were evaluated in the livers of STAM mice at 10 weeks of age. **(E)** The total NASH score was calculated by combining the sum of steatosis score, inflammation score, and ballooning score (n=6/group). **(F)** Oil-red stained liver tissues of STAM mice with or without *A muciniphila* adminstration. Scale bars, 50 µm. The serum ALT **(G)**, AST, triglyceride, blood glucose and GLP-1 **(H-K)** levels were measured. Data represent mean ± SEM of two pooled experiments. *P<0.05 vs STAM group by unpaired Student’s t test. ns, not significant. AKK, *Akkermansia muciniphila*; ALT, alanine aminotransferase; AST, aspertate aminotransferase; GLP-1, glucagon-like peptide-1.

**Figure 4:**
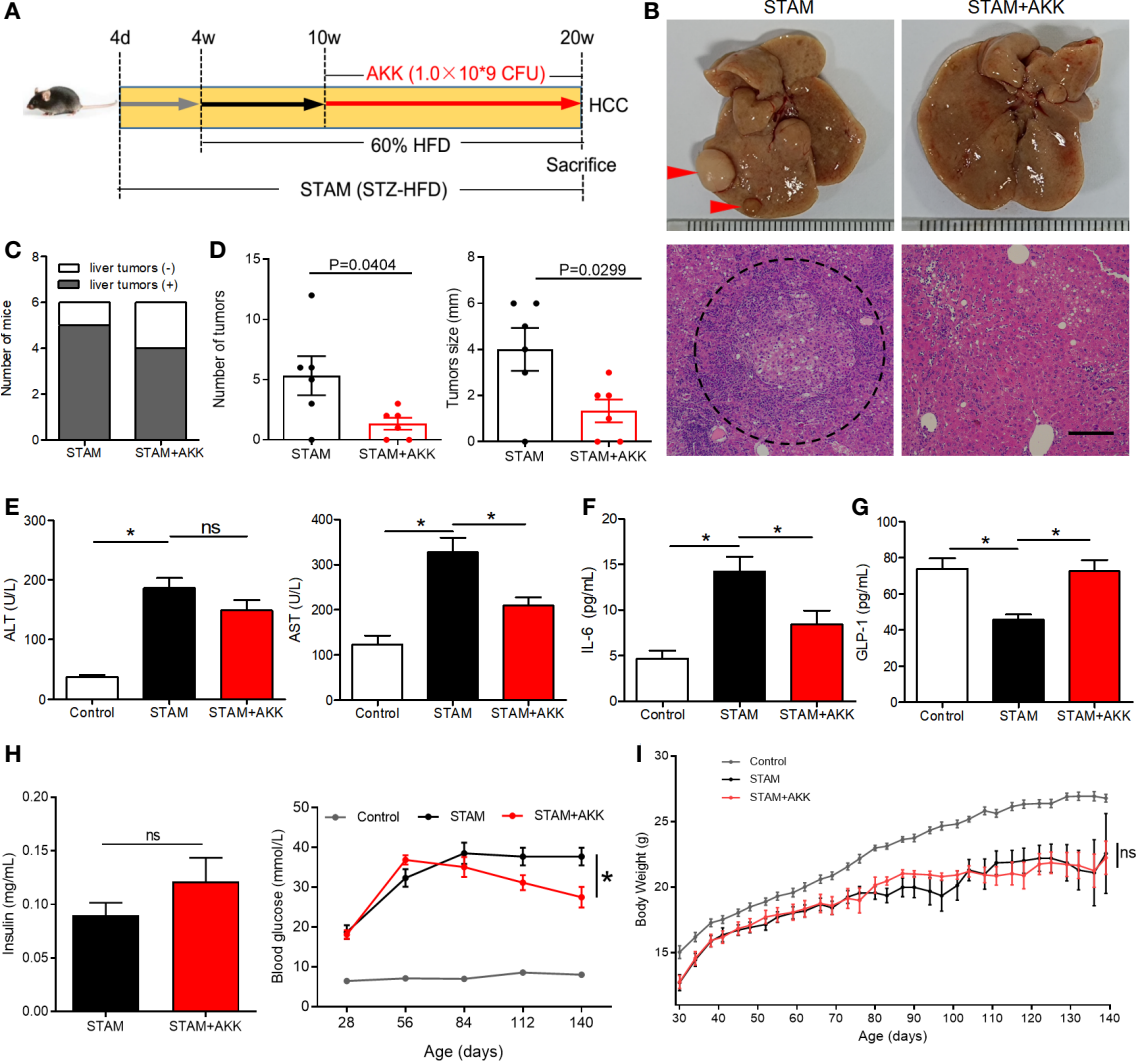
*A muciniphila* administration blunted hepatocarcinogenesis in STAM mice. **(A)** Experimental design and protocol. STAM mice were treated with *A muciniphila* or saline (Control) starting at 10 weeks of age for 10 weeks, and were killed at 20 weeks of age (n=6/group). **(B)** Macroscopic image of the liver surface (arrowheads: tumor) and histological images of liver tissues by H&E staining (dashed circle: tumor). Scale bars, 200 µm. **(C)** The total number of tumor nodules on the liver surface and the maximum diameter of the tumor nodules were compared between the groups. **(D)** The number of mice developed with liver tumor were counted. The serum levels of **(E)** ALT, AST, **(F)** GLP-1, **(G)** IL-6 and **(H)** insulin of the STAM mice (20 weeks of age) were measured. **(H)** The blood glucose were recorded every week. **(I)** The body weight was recorded every 4 days. Data represent mean ± SEM of two pooled experiments. *P<0.05 by unpaired Student’s t test. ns, not significant. STZ, streptozotocin; STAM, streptozotocin + high fat diet-treated mice; AKK, *Akkermansia muciniphila*; AST, aspertate aminotransferase; ALT, alanine aminotransferase; GLP-1, glucagon-like peptide-1.

**Figure 5:**
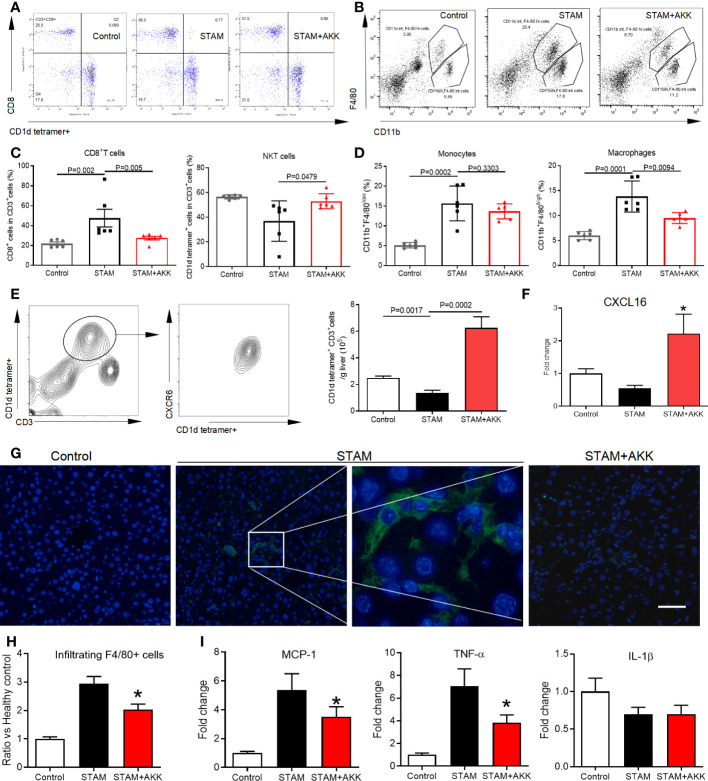
*A muciniphila* increases NKT cell but reduces macrophage infiltration and inflammatory cytokines in the livers of STAM. **(A)** Quantification of subgroups of T cells and **(B)** macrophages by flow cytometry. The percent of **(C)** CD8^+^ T cells, NKT cells, **(D)** monocytes and macrophages in the liver were evaluated. **(E)** Representative CXCR6 staining in hepatic NKT cells from three independent experiments. Relative mRNA levels of **(F)** CXCL16 in the liver were determined by real-time PCR and normalized to GAPDH. Data represent mean ± SEM of two pooled experiments (n=6/group). **(G)** Immunofluorescent staining for macrophages (Scale bars, 50 µm). **(H)** The infiltration macrophages in the liver were counted (vs healthy control group). **(I)** Relative mRNA levels of MCP-1, TNF-α and IL-1β in the liver were determined by real-time PCR and normalized to GAPDH. Data represent mean ± SEM of two pooled experiments (n=6/group). AKK, *Akkermansia muciniphila*. MCP-1, monocyte chemoattractant protein-1. *P<0.05 vs STAM group by unpaired Student’s t test.

**Figure 6:**
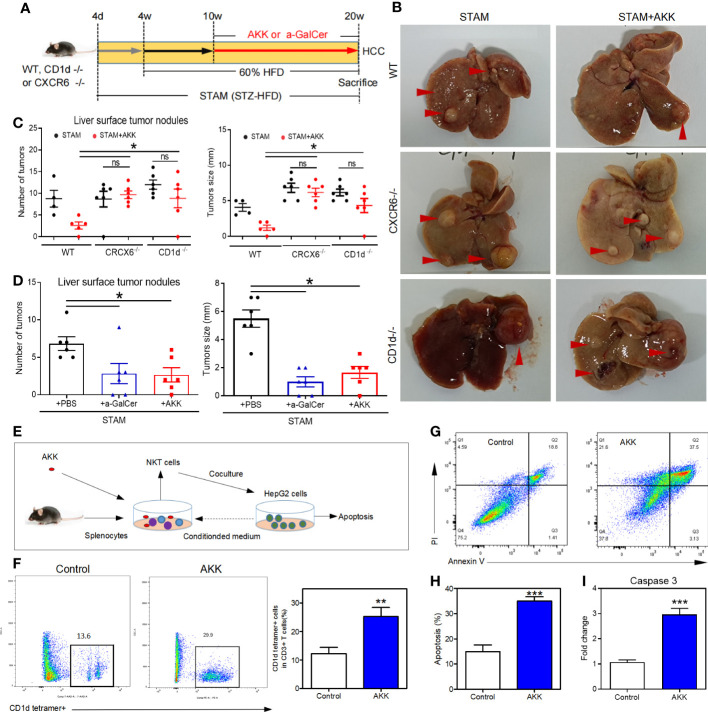
*A muciniphila* inhibit NASH-related HCC through NKT cells. **(A)** Experimental design and protocol. CD1d^−/−^ (n=6), CXCR6^−/−^ (n=6) or wild-type mice (n=4 or 5) were treated with *A muciniphila*, a-GalCer (postive control) or saline (placebo-control) for 10 weeks starting at 10 weeks of age twice a day. Mice were killed at 20 weeks of age. **(B)** Macroscopic images of the liver surface (arrowheads: tumor). **(C, D)** The total number of tumor nodules on the liver surface were counted and the maximum diameter of the tumor nodules in the liver were measured. **(E)** Experimental design and protocol of *in vivo* experiments. **(F)** The fraction of NKT cells in the splenocytes was cultured with HepG2 cell conditioned medium with or without *A muciniphila* for 72 hours. **(G, H)** The percentage of apoptotic HepG2 cells was measured by flow cytometry. Data represent mean ± SEM of two pooled experiments. **(I)** The mRNA expression of caspase 3 in HepG2 cells was detected by qPCR. *P < 0.05. **p<0.001, ***p<0.0001 by unpaired Student’s t test. STAM, streptozotocin + high fat diet-treated mice; AKK, Akkermansia muciniphila; ns, not significant.

The authors apologize for this error and state that this does not change the scientific conclusions of the article in any way. The original article has been updated.

