# Corrigendum: Akkermansia muciniphila suppressing nonalcoholic steatohepatitis associated tumorigenesis through CXCR6^+^ natural killer T cells

**DOI:** 10.3389/fimmu.2023.1297103

**Published:** 2023-11-27

**Authors:** Tao Li, Xinlong Lin, Binhai Shen, Wujian Zhang, Yangyang Liu, Hongbin Liu, Ye Wang, Lijun Zheng, Fachao Zhi

**Affiliations:** ^1^ Guangdong Provincial Key Laboratory of Gastroenterology, Department of Gastroenterology, Institute of Gastroenterology of Guangdong Province, Nanfang Hospital, Southern Medical University, Guangzhou, China; ^2^ Department of General Surgery of the First Affiliated Hospital of Heilongjiang University of Traditional Chinese Medicine, Haerbin, China; ^3^ Guangzhou ZhiYi Biotechnology Co. Ltd., Guangzhou, China

**Keywords:** cancer progression, *Akkermansia muciniphila*, tumor immune surveillance, nonalcoholic fatty liver disease, hepatocellular carcinoma - metabolic syndrome - non-alcoholic fatty liver disease (NAFLD) - non-alcoholic steatohepatitis - NASH-HCC

In the published article, there was an error in **Figure 1F** as published. According to the experimental design of this paper, the original purpose of **Figure 1F** was to confirm the decreased abundance of *A. muciniphila* in the colon of STAM mice (20 weeks old) compared to control mice. However, in **Figure 1F**, we included FISH results of STAM mice at 4, 10, 16 weeks of age by mistake, which was not consistent with the description in the Results part. According to the description in the Results part, the comparison of the FISH results of STAM mice (20 weeks old) with control mice should be include in **Figure 1F**, which makes the results more readable and rigorous. The corrected **Figure 1F** and its corrected figure legend appear below.

**Figure 1:**
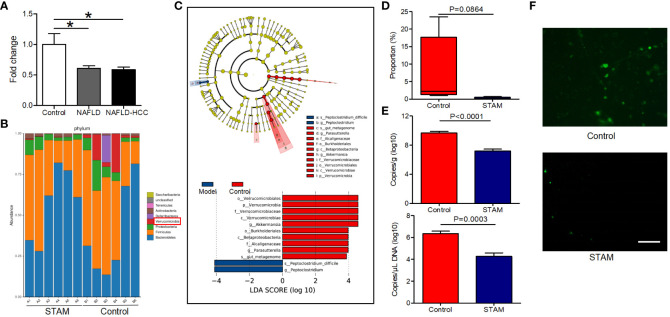
The intestinal abundance of *A muciniphila* was decreased in patients and mice with NAFLD-HCC (STAM at 20 weeks) **(A)** The relative abundance of *A muciniphila* in patients with NAFLD or NAFLD-HCC and healthy controls by qPCR (n=6). *P<0.05 by unpaired Student’s t test. **(B)** Comparison of the faecal microbiota between STAM at 20 weeks and controls at the species level by 16S rRNA sequencing. **(C)** LEfSe analysis of the faecal microbiota between the STAM at 20 weeks and healthy controls. Cladogram displays the taxonomic tree of differentially abundant taxa. Histogram represents the LDA scores of bacteria with significant differential abundance between the compared groups, identified by different colors. **(D)** The proportion of *Akkermansia* in the faecal microbiota was compared. **(E)** qPCR validation of the abundance of *A muciniphila* in STAM at 20 weeks and control. **(F)** FISH detection of *A. muciniphila* on the surface of the colon from STAM mice at 20 weeks of age and control mice. Data are presented as the mean ± SEM and were analysed by unpaired Student’s t test. NAFLD, non-alcoholic fatty liver disease; HCC, hepatocellular carcinoma; STAM, streptozotocin+high fat diet-treated mice.

The authors apologize for this error and state that this does not change the scientific conclusions of the article in any way. The original article has been updated.

