# Supplementary figures and images for: Akkermansia muciniphila suppressing nonalcoholic steatohepatitis associated tumorigenesis through CXCR6+ natural killer T cells
Source: Front Immunol. 2022 Dec 1;13:1047570. doi: 10.3389/fimmu.2022.1047570 (PMC9755844; doi:10.3389/fimmu.2022.1047570)

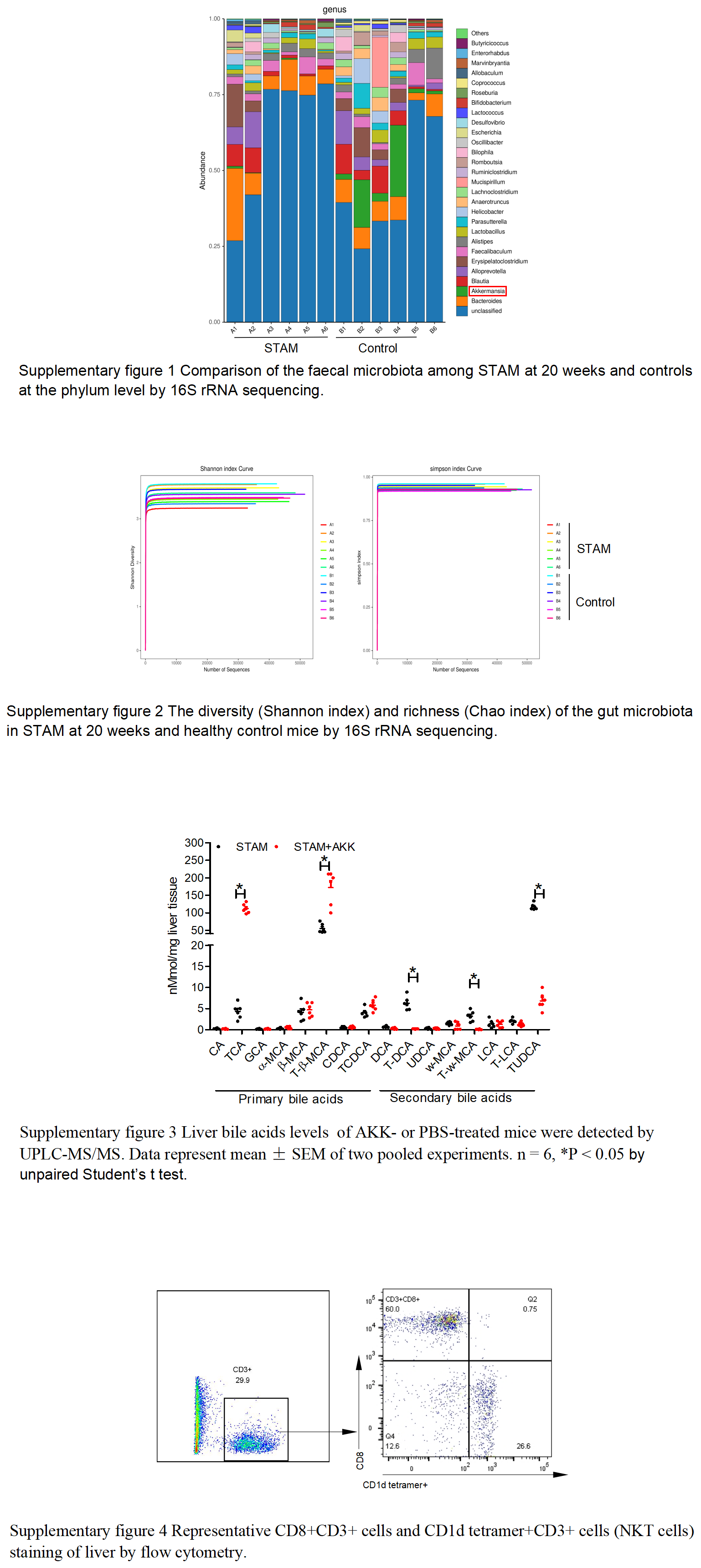

Supplement: Supplementary file 1 [file Image_1.tif]
