# Supplementary material for: Akkermansia muciniphila suppressing nonalcoholic steatohepatitis associated tumorigenesis through CXCR6+ natural killer T cells
Source: Front Immunol. 2022 Dec 1;13:1047570. doi: 10.3389/fimmu.2022.1047570 (PMC9755844; doi:10.3389/fimmu.2022.1047570)
Supplement: Supplementary file 2 [file Table_1.docx]

Supplementary table 1. Demographic and clinical characterization of study groups

| Characteristics | Control  (n=6) | NAFLD  (n=6) | NAFLD-HCC  (n=6) |
| --- | --- | --- | --- |
| Male (%) | 50 | 50 | 50 |
| Age (years) | 56.1±2.2 | 55.8±2.4 | 67.5±2.7 |
| BMI (kg/m^2^) | 20.0±0.6 | 27.5±1.0 | 26.7±1.0 |
| ALT (U/L) | 18.0±1.4 | 41.7±1.7 | 72.3±5.5 |
| AST (U/L) | 21.2±1.3 | 32.6±2.6 | 80.3±9.6 |
| Glucose (fasting; mg/dL) | 76.8±4.6 | 102.2±4.1 | 129.8±6.8 |
| NAFLD: nonalcoholic steatohepatitis; HCC: Hepatocellular carcinoma; BMI: Body Mass Index; ALT: alanine transaminase; AST: aspartate aminotransferase; | | | |
