# Supplementary material for: Akkermansia muciniphila suppressing nonalcoholic steatohepatitis associated tumorigenesis through CXCR6+ natural killer T cells
Source: Front Immunol. 2022 Dec 1;13:1047570. doi: 10.3389/fimmu.2022.1047570 (PMC9755844; doi:10.3389/fimmu.2022.1047570)
Supplement: Supplementary file 4 [file DataSheet_1.pdf]

Supplementary Methods

Fluorescent in situ hybridisation (FISH)

Localisation of A. muciniphila on the surface of the colon from NAFLD mice was performed by fluorescent in situ hybridisation (FISH) as described previously [1]. The fluorescein isothiocyanate (FITC)-labelled A. muciniphila probe (FITC-CCTTGCGGTTGGCTTCAGAT) was from GENERAY Biotechnology (Shanghai, China). Before applying the hybridization solution(100mM Tris [pH 7.2], 0.9M NaCl, 0.1% sodium dodecyl sulfate), the sections were circumscribed with a hydrophobic PAP-pen. In a dark and moisture room, the sample were hybridized in 40 mL of hybridization buffer with 200 ng A. muciniphila probe for 16 hours. Hybridization buffer and washing solution (100mM Tris [pH 7.2], 0.9M NaCl) were prewarmed (45°C). After that, samples were washed in prewarmed hybridization buffer (150mL) for 15 minutes and subsequently in prewarmed washing solution (150mL) for another 15 minutes. The samples were rinsed in water, air dried and mounted in Vectashield for epifluorescence microscopy. Hybridised samples were imaged with a Zeiss LSM710 confocal microscope (Carl Zeiss, Germany).

Biochemical profiles and ELISA

Fasting blood glucose was measured using dynamic type medical test blood glucometer (ROCHE, Germany). Serum alanine aminotransferase (ALT) and aspartate aminotransferase (AST) were measured by Mouse Aspartate ALT and AST ELISA kit. To measure serum insulin concentration, blood samples were leave to rest for 30 min at room temperature. After the blood clotted completely, the serum was separated by centrifugation at 4,000g for 10 min and stored at −80 °C. Serum insulin concentration was measured using an Ultra-Sensitive Mouse Insulin ELISA Kit (Morinaga Institute of Biological Science, Yokohama, Japan). For the measurement of plasma GLP-1, mice were fasted for 5 h in the morning. Blood samples were obtained by retro-orbital sinus puncture 0 min and 10 min after oral gavage of 2 g /kg body mass glucose and collected into prechilled EDTA-coated tubes containing 1 μg/ml diprotin A (6019; Tocris Bioscience), centrifuged without delay, and the plasma was then separated and stored at −80 °C. GLP-1 concentration was determined using the GLP-1 ELISA kit (RayBiotech) as previously [2].

Histology and immunohistochemistry (IHC)

For histology assessment, colon tissue sections were fixed, dehydrated, embedded and sectioned (5 μm) for IHC assays, the paraffin sections were deparaffinised, endogenous enzymes were inactivated and antigens were thermally repaired. The macrophage staining was done as previously [3].

Patients and sample collection

Patients who were diagnosed with NAFLD or NAFLD-HCC were enrolled in the study after obtaining their informed consent. Patients were clinically diagnosed with liver diseases, meeting the criteria of NAFLD: fatty liver from

imaging (ultrasound or tomography) or from biopsy according to NAFLD activity score. HCC was diagnosed according to international guidelines, integrating history, physical examination, biochemistry, and imaging techniques obtained by multiphasic CT, and/or dynamic contrast-enhanced MRI,[24] and further confirmed by histopathological examination of surgical resection specimens. Exclusion criteria included: age <18 years; men who consumed ≥30 g and women who consumed >10 g of alcohol; other causes of HCC (including viral, alcoholic, autoimmune, cholestatic liver diseases, inherited liver diseases, etc.); other primary liver cancers (e.g. mixed HCC and cholangiocarcinoma); consumption of antibiotics, probiotics or any other medications that may have an effect on steatohepatitis within 3 months before recruitment; known gastrointestinal disease; previous gastrointestinal surgery; regular proton-pump inhibitor or lactulose therapy. *A. muciniphila* abundance was quantified in stool samples of patients with NAFLD (n=6), NAFLD-HCC (n=6) and in non-obese healthy controls (n=6). Demographic characteristics of study subjects were shown in supplementary table 1. The stool samples were collected from patients in the First Hospital of Harbin Medical University from December 2020 to May 2022 and immediately frozen and stored at −80°C.

16S rRNA sequencing and analysis

Sequencing was as previously described by Zhang et al [4]. DNA was extracted from collected stool samples and 16S V3-V4 region amplification were performed on the liquid hand ling robots. After genomic DNA extraction, PCR amplification, the DNA amplicons are linearized as single-stranded. Adding modified DNA polymerase and four fluorescently labeled dNTP to only one base in each cycle. Scan the reaction plate surface by laser and read the nucleotide species polymerized in the first round of reaction of each template sequence. The "fluorescent group" and the "termination group" are chemically cut to restore the 3'end viscosity, and continue to polymerize the second nucleotide. The fluorescence signal results collected in each round were counted, and the sequence of the template DNA fragment was obtained. The sequencing PEreads first distinguished each sample according to barcode, then the sequence quality was controlled and filtered, and then spliced according to the overlap relationship. The assembled sequences quality were controlled and filtered again, and finally the optimized sequence was obtained. Optimize sequence for OTU cluster analysis and species taxonomic analysis. Based on OTU cluster analysis results, we can perform multiple diversity index analysis (alpha diversity analysis within samples) and sequencing depth detection; based on taxonomic information, we can perform statistical analysis of community structure at each classification level, and beta diversity analysis between samples. Based on the above analysis, a series of in-depth statistical and visual analyses of community structure and phylogeny can be performed.

Real-time PCR

Total cellular RNA was isolated using the RNAiso Plus Kit (Takara). Purified RNA was reversely to cDNA synthesis using Quantitect Reverse Transcription Kit (Qiagen) according to the manufacturer's protocol. Real-time PCR was

performed in triplicate using the LightCycler 480 System (Roche). Data were generated and analyzed using SDS 2.4 and RQ manager 1.2 software. For murine samples, mRNA expression levels were normalized to the housekeeping gene β-actin. Primer sequence:

TNF-α forward AAGGGAGAGTGGTCAGG- and reverse TCTGTGAGGAAGGCT

CXCL16 forward AACCAGGGCAGTGTCGC and reverse AGGCAAATGTTTTTGGTGG

MCP-1 forward TAAAAACCTGGATCGGAACCAA and reverse ATTTTGGACCTAGCCTTGGTT

IL-1β forward GCAACTGTTCCTGAACTCAACT and reverse ATCTTTTGGGGTCCGTCAACT.

References:

1. Boye M, Aalbaek B, Agerholm JS. Fusobacterium necrophorum determined as abortifacient in sheep by laser capture microdissection and fluorescence in situ hybridization. Mol Cell Probes 2006;20:330-6.
2. Yoon HS, Cho CH, Yun MS, Jang SJ, You HJ, Kim JH, et al. Akkermansia muciniphila secretes a glucagon-like peptide-1-inducing protein that improves glucose homeostasis and ameliorates metabolic disease in mice. Nat Microbiol 2021;6:563-573.
3. van der Windt DJ, Sud V, Zhang H, Varley PR, Goswami J, Yazdani HO, et al. Neutrophil extracellular traps promote inflammation and development of hepatocellular carcinoma in nonalcoholic steatohepatitis. Hepatology 2018;68:1347-1360.
4. Zhang W, Zhu B, Xu J, Liu Y, Qiu E, Li Z, et al. Bacteroides fragilis Protects Against Antibiotic-Associated Diarrhea in Rats by Modulating Intestinal Defenses. Front Immunol 2018;9:1040.
